# Supplementary material for: SHLD2/FAM35A co‐operates with REV7 to coordinate DNA double‐strand break repair pathway choice
Source: EMBO J. 2018 Aug 28;37(18):e100158. doi: 10.15252/embj.2018100158 (PMC6138439; doi:10.15252/embj.2018100158)
Supplement: Supplementary file 1 — Expanded View Figures PDF [file EMBJ-37-e100158-s001.pdf]

Expanded View Figures

Figure EV1. Analysis of REV7 interactome.

A Schematic representation of AP-MS stable interaction Flag-REV7 pull-down and the BioID BirA*-REV7 biotinylation of proximal interactors.
B HEK293-TREx cells stably expressing an inducible Flag-REV7 construct were tested for expression following induction with tetracycline as indicated. After lysis, samples were immunoblotted for FLAG and REV7. Actin was used as a loading control.
C HEK293-TREx cells stably expressing an inducible BirA-Flag or BirA-Flag-REV7 construct were tested for expression and biotinylation following induction with tetracycline and incubation with biotin as indicated. After lysis, samples were immunoblotted for FLAG and streptavidin. Actin was used as a loading control.
D The interactome of REV7 obtained from both the AP-MS and the BioID approaches was analyzed for pathway enrichment using EnrichR. The *y*-axis represents the ratio of the number of genes from the dataset that map to the pathway and the number of all known genes ascribed to the pathway and is defined as enrichment of *P*-value (−log10).
E Network representation of the selected 11 high-confidence interactors of REV7 (annotated as MAD2L2 in this figure) and their previously described interactors. Proteins are represented following the k-means clustering through STRING v10.5.
F Cell cycle distribution of U2OS EJ5-GFP cells transfected with the indicated siRNAs and subsequently for propidium iodide (PI) staining and flow cytometry analysis. Data are presented as the mean (n = 2).

© 2018 The Authors

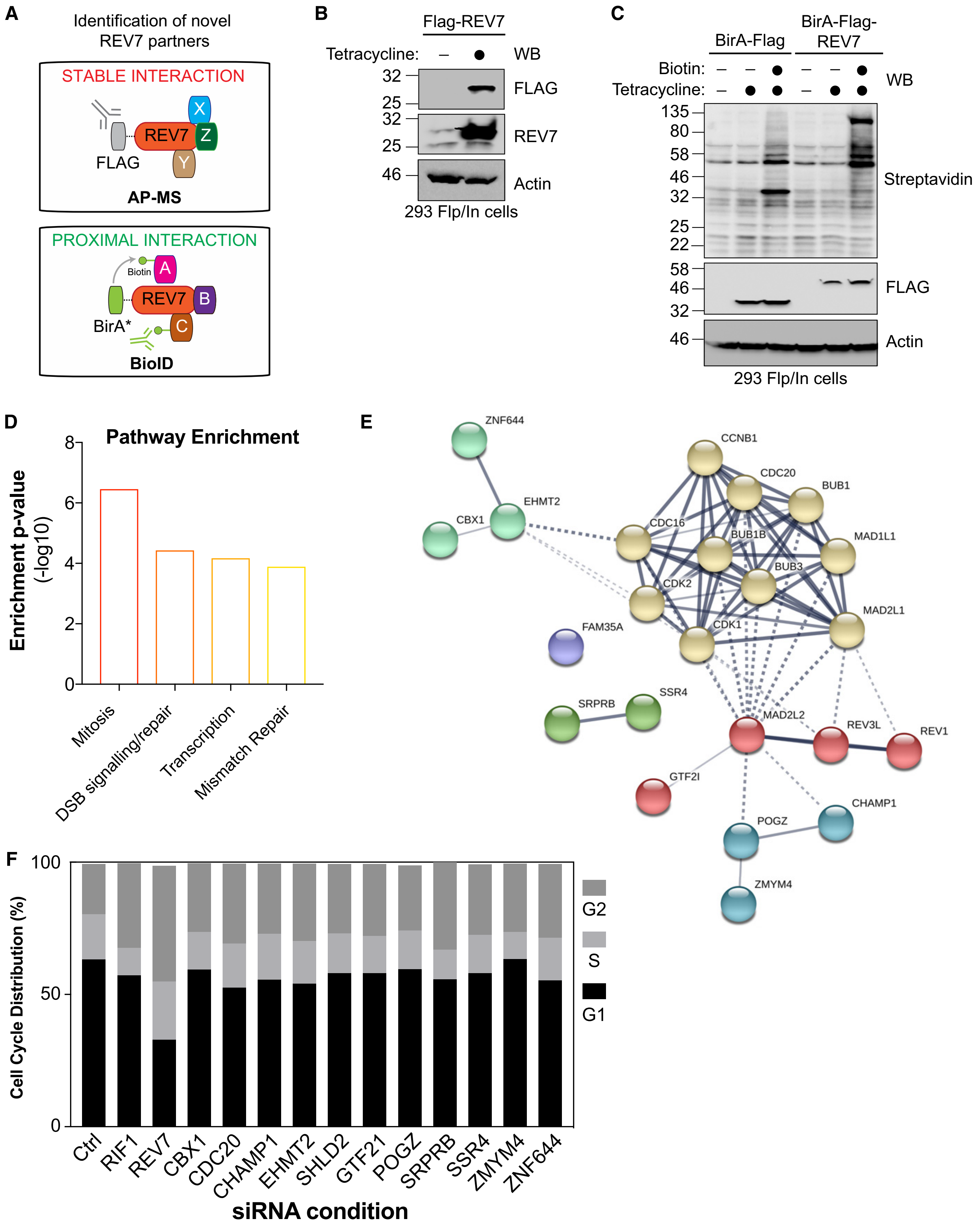

Figure EV1.

© 2018 The Authors

Figure EV2. Evaluation of the impact of SHLD2 on DNA repair.

A U2OS and MCF-7 cells were subjected to lentiviral-mediated short hairpin RNA knockdown for REV7 or SHLD2, selected with puromycin for 48 h then harvested. Total RNA was isolated, cDNA was generated, and levels of REV7 and SHLD2 were identified by qPCR. mRNA levels were normalized to mRNA levels of GAPDH. Data are presented as the mean ± SEM (n = 3). Significance was determined by one-way ANOVA followed by a Dunnett's test. *$P < 0.0005$.

B Representative images of Comet Assay Tail Moment quantified in Fig 2. U2OS cells stably expressing shCtrl, shREV7, or shSHLD2 were exposed to irradiation (10 Gy) and run in low melting agarose under neutral conditions. Immunofluorescence against DNA stained with SYBR Gold was performed to measure the tail moment. Scale bar = 10 μm.

C U2OS cells were transfected with small interfering RNA against REV7 or SHLD2 for 48 h, total RNA was isolated, cDNA was generated, and levels of REV7 and SHLD2 were identified by qPCR. mRNA levels were normalized to mRNA levels of GAPDH. Data are presented as the mean ± SEM (n = 3). Significance was determined by one-way ANOVA followed by a Dunnett's test. *$P < 0.0005$.

D Representative flow cytometry profiles of U2OS cells transfected with the indicated siRNA and subsequently treated with NCS for 30 min before being trypsinized and processed for anti-γ-H2AX (y-axis) and PI (x-axis) staining.

© 2018 The Authors

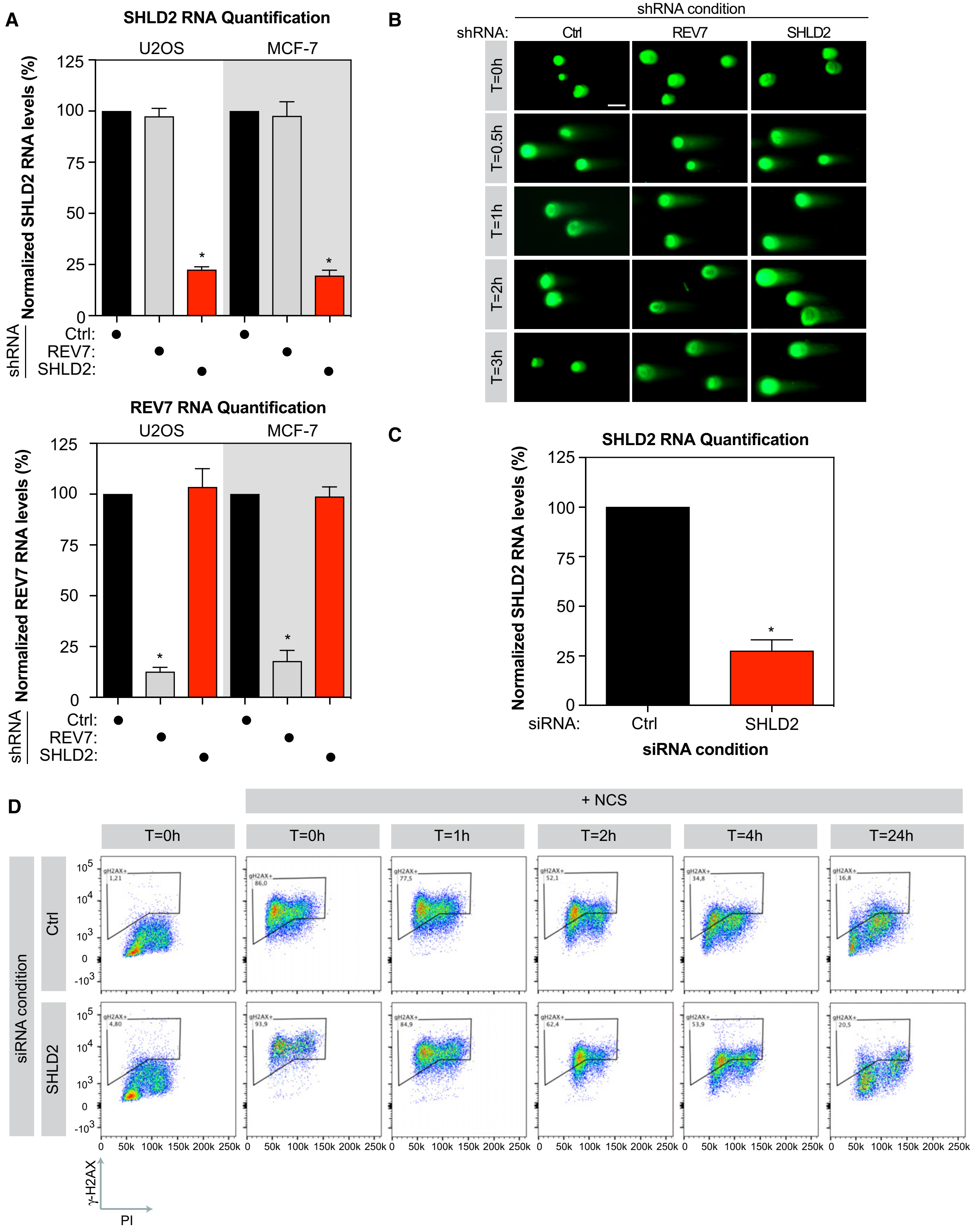

Figure EV2.

© 2018 The Authors

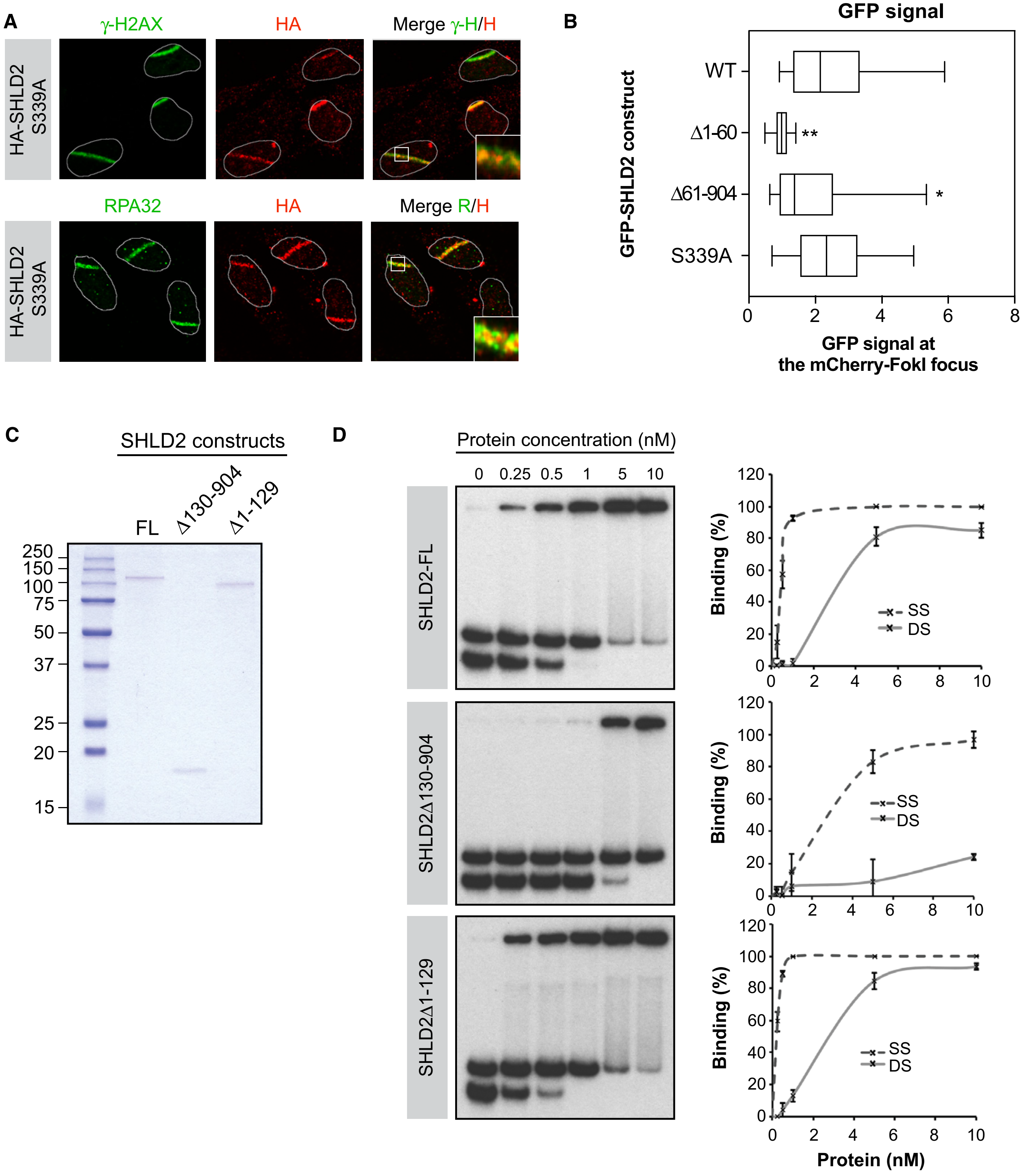
A
γ-H2AX
HA
Merge γ-H/H
HA-SHLD2 S339A
RPA32
HA
Merge R/H
HA-SHLD2 S339A
B
GFP signal
GFP-SHLD2 construct
WT
Δ1-60
**
Δ61-904
*
S339A
0
2
4
6
8
GFP signal at the mCherry-FokI focus
C
SHLD2 constructs
FL
Δ130-904
Δ1-129
250
150
100
75
50
37
25
20
15
D
Protein concentration (nM)
0
0.25
0.5
1
5
10
SHLD2-FL
SHLD2Δ130-904
SHLD2Δ1-129
Binding (%)
SS
DS
Protein (nM)

Figure EV3.

Figure EV3. Structure-function analysis of SHLD2.

A U2OS cells stably expressing HA-SHLD2-S399A were processed as previously described. Immunofluorescence against endogenous HA, γ-H2AX (Top), and RPA32 (Bottom) epitope was subsequently performed to monitor their accumulation at sites of damage. Shown are representative micrographs.

B U2OS LacR-Fok1 cells were transfected with GFP-SHLD2, GFP-SHLD2-S339A, GFP-SHLD2-Δ1-60, or GFP-SHLD2-Δ61-904 mutant, and 24 h later, DNA damage was induced using Shield-1 and 4-OHT. The cells were then processed for GFP and mCherry immunofluorescence. Shown is the quantification of cells expressing GFP at Fok1 sites. Data are represented as a box-and-whisker plot where the whiskers represent the 10–90 percentile. At least 75 cells were counted per condition. Significance was determined by one-way ANOVA followed by a Dunnett's test. *$P < 0.005$, **$P < 0.0005$

C Recombinant SHLD2 constructs were purified from Sf9 insect infected, and protein purity was assessed by Coomassie Blue stain. Shown are the protein samples used for the DNA-binding assay.

D *In vitro* DNA-binding assay was performed using a purified recombinant SHLD2 or SHLD2-mutants (concentration range: 0–10 nM) with ^{32}P labeled DNA oligonucleotide substrates. Protein–DNA complexes were subjected to electrophoresis and visualized by autoradiography. Representative binding experiments (left panel; $n = 3$) and quantification of the binding efficiency (right panel) are shown.

Figure EV4. Characterization of the role of SHLD2 in the NHEJ pathway.

A U2OS LacR-Fok1 cells were transfected with small interfering RNA directed against 53BP1, RIF1, REV7, or BRCA1. Forty-eight hours later, the cells were then processed for AF647 and mCherry immunofluorescence. Shown is the ratio of fluorescence of AF647 at Fok1 focus compared to background. Data are represented as a box-and-whisker plot where the whiskers represent the 10–90 percentile. At least 75 cells were counted per condition. Significance was determined by one-way ANOVA followed by a Dunnett's test. *$P < 0.005$

B U2OS LacR-Fok1 cells were transfected with small interfering RNA directed against SHLD2. 48 h later, DNA damage was induced using Shield-1 and 4-OHT, followed by staining to identify 53BP1, RIF1, REV7, or BRCA1 protein localization by indirect AF647 fluorescence. The cells were then processed for AF647 and mCherry immunofluorescence. Shown is the quantification of cells expression GFP at Fok1 sites. Data are represented as a box-and-whisker plot where the whiskers represent the 10–90 percentile. At least 75 cells were counted per condition. Significance was determined by one-way ANOVA followed by a Dunnett's test.

C EJ5-2OS cells were transfected with small interfering RNA against REV7 or SHLD2 for 48 h, total RNA was isolated, cDNA was generated, and levels of REV7 and SHLD2 were identified by qPCR. mRNA levels were normalized to mRNA levels of GAPDH. Data are presented as the mean ± SEM ($n = 3$). Significance was determined by one-way ANOVA followed by a Dunnett's test.

D CH12F2-3 cells were subjected to lentiviral-mediated short hairpin RNA knockdown for REV7 or SHLD2, selected with puromycin for 48 h then harvested. Total RNA was isolated, cDNA was generated, and levels of REV7 and SHLD2 were identified by qPCR. mRNA levels were normalized to mRNA levels of GAPDH. Data are presented as the mean ± SEM ($n = 3$). Significance was determined by one-way ANOVA followed by a Dunnett's test.

E Proliferation of the different transduced CH12F3-2 cell lines was monitored using CFSE dilution. FACS profiles are representative of three independent experiments.

F Schematic diagram of both the DR-GFP reporter assay (Top) and the SA-GFP reporter assay showing (Bottom).

© 2018 The Authors

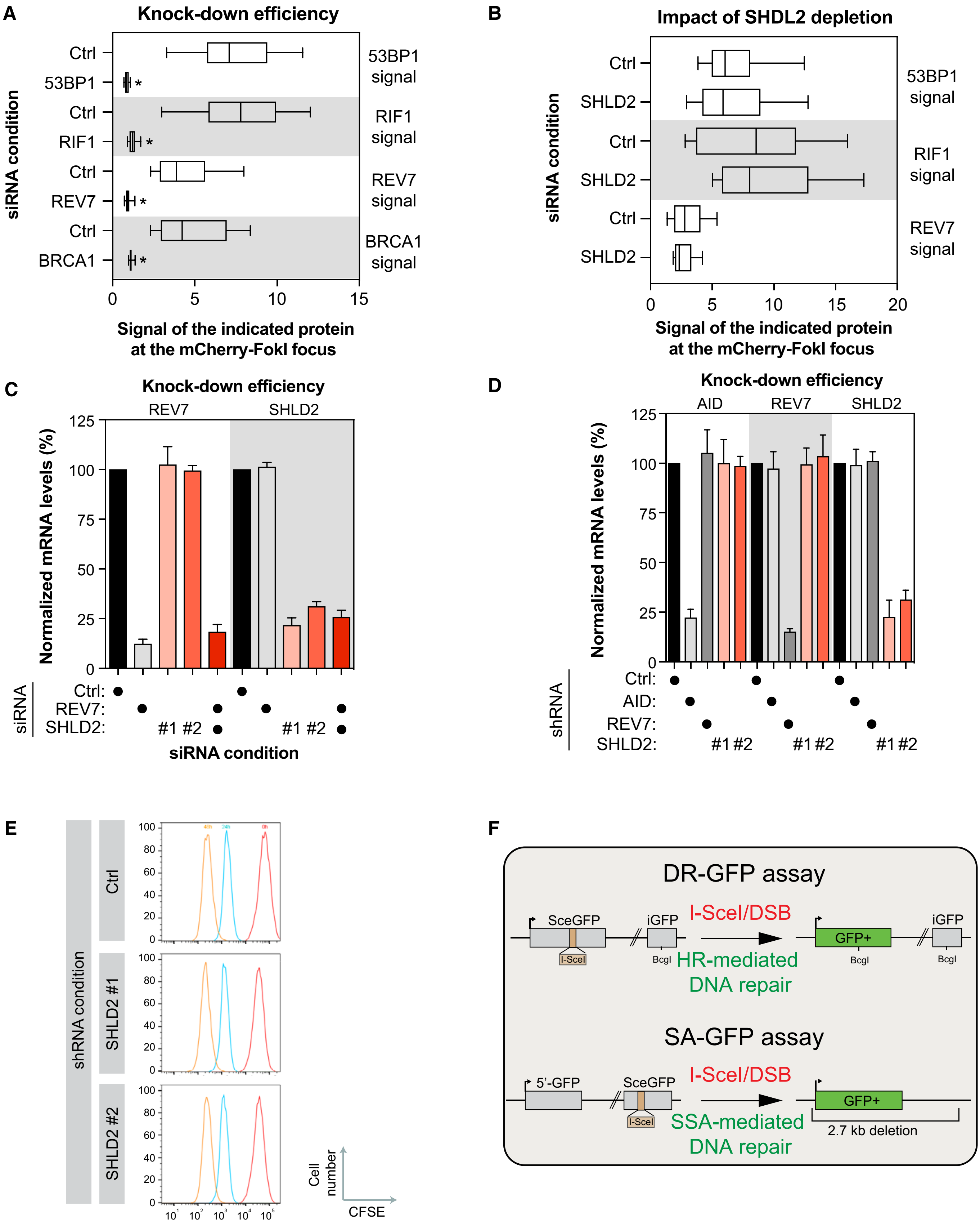
A
Knock-down efficiency
siRNA condition
Ctrl
53BP1
Ctrl
RIF1
Ctrl
REV7
Ctrl
BRCA1
53BP1 signal
RIF1 signal
REV7 signal
BRCA1 signal
Signal of the indicated protein at the mCherry-FokI focus
B
Impact of SHDL2 depletion
Ctrl
SHLD2
53BP1 signal
RIF1 signal
REV7 signal
C
Knock-down efficiency
REV7
SHLD2
Normalized mRNA levels (%)
siRNA
Ctrl:
REV7:
SHLD2:
#1 #2
siRNA condition
D
Knock-down efficiency
AID
REV7
SHLD2
shRNA
Ctrl:
AID:
REV7:
SHLD2:
#1 #2
E
shRNA condition
Ctrl
SHLD2 #1
SHLD2 #2
Cell number
CFSE
F
DR-GFP assay
SceGFP
iGFP
I-SceI/DSB
HR-mediated DNA repair
GFP+
I-SceI
BcgI
SA-GFP assay
5'-GFP
SSA-mediated DNA repair
2.7 kb deletion

Figure EV4.

Figure EV5. Characterization of the role of SHLD2 in the HR pathway.

A HeLa DR-GFP cells were transfected with the indicated siRNAs. At 24 h post-transfection, cells were transfected with the I-SceI expression plasmid, and the GFP^+ population was analyzed 48-h post-plasmid transfection. The percentage of GFP^+ cells was determined for each individual condition and subsequently normalized to the non-targeting condition (siCTRL). Data are presented as the mean ± SD (n = 3). Significance was determined by one-way ANOVA followed by a Dunnett's test. *$P < 0.0005$, **$P = 0.0001$.

B U2OS SA-GFP cells were transfected with the indicated siRNAs. At 24 h post-transfection, cells were transfected with the I-SceI expression plasmid, and the GFP^+ population was analyzed 48-h post-plasmid transfection. The percentage of GFP^+ cells was determined for each individual condition and subsequently normalized to the non-targeting condition (siCTRL). Data are presented as the mean ± SD (n = 3). Significance was determined by one-way ANOVA followed by a Dunnett's test. *$P < 0.05$, **$P < 0.005$, ***$P < 0.0005$.

C Representative images of the DNA fiber assay obtained from U2OS cells which were transfected with the indicated siRNAs and then treated with CldU, IdU, and NCS 48 h post-transfection as indicated. The slides were stained, dehydrated, mounted, and visualized and shown is the quantification of CldU/IdU tract length in order to visualize DNA end resection.

D U2OS cells were transfected with the indicated siRNAs. The DNA damage experimental condition was performed using 500 ng/ml of neocarzinostatin (NCS) for 1 h. Cells were washed in D-PBS and harvested with a lysis buffer [50 mM HEPES, KOH (pH 8.0), 100 mM KCl, 2 mM EDTA, 0.1% NP-40, 10% glycerol] supplemented with protease/phosphatase inhibitors. The resulting whole cell lysates were analyzed by immunoblotting using p-RPA and α-tubulin, then analyzed by immunoblotting using GFP, REV7, and p-Chk1 antibodies.

E HeLa DR-GFP cells were co-transfected with siBRCA1 and the indicated siRNAs. At 24 h post-transfection, cells were transfected with the I-SceI expression plasmid, and the GFP^+ population was analyzed 48-h post-plasmid transfection. The percentage of GFP^+ cells was determined for each individual condition and subsequently normalized to the non-targeting condition (siCTRL). Data are presented as the mean ± SD (n = 3). Significance was determined by one-way ANOVA followed by a Dunnett's test. *$P < 0.05$, **$P < 0.0005$.

 © 2018 The Authors

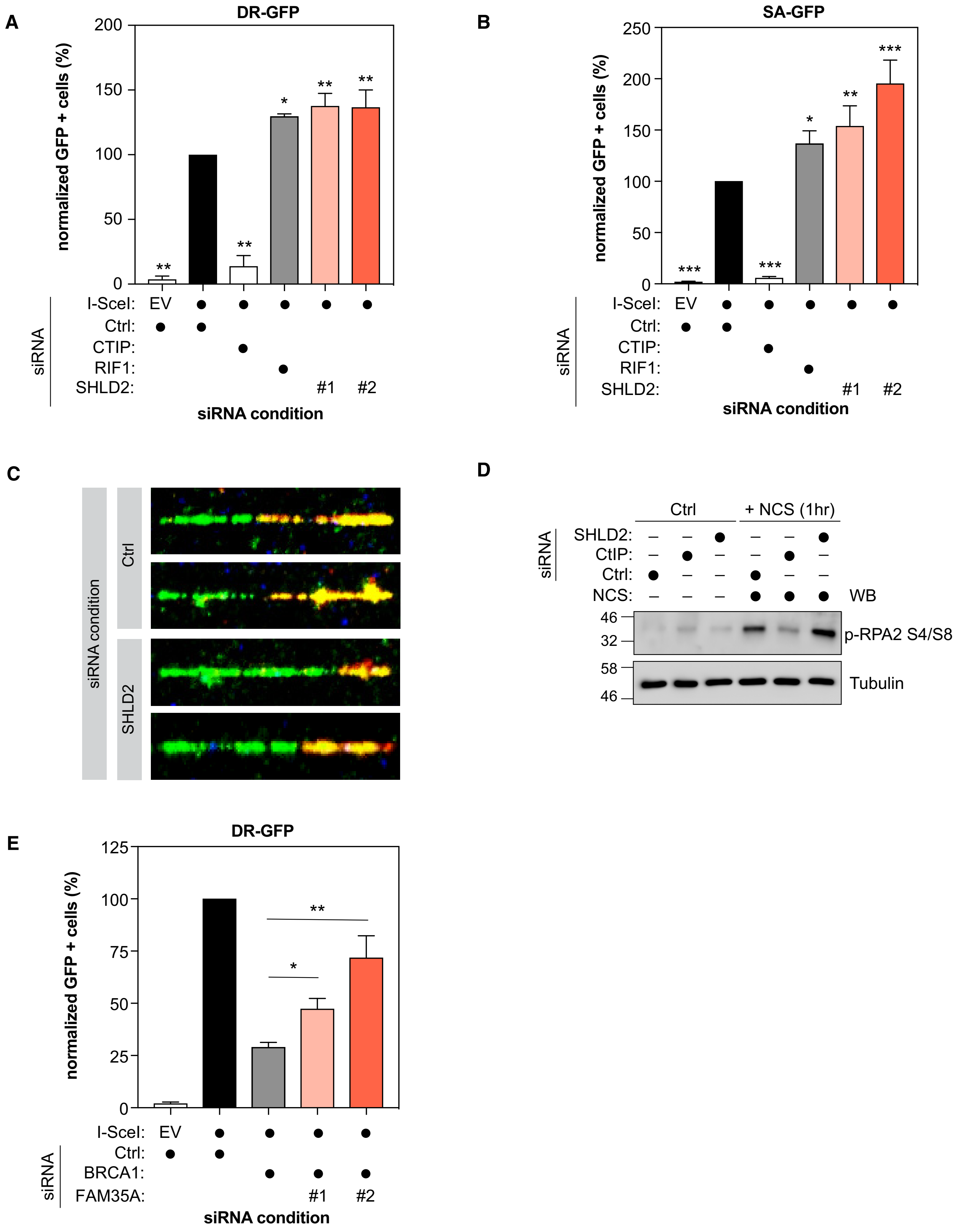
A
DR-GFP
normalized GFP + cells (%)
200
150
100
50
0
**
**
*
**
**
I-SceI: EV
Ctrl:
CTIP:
RIF1:
SHLD2: #1 #2
siRNA
siRNA condition
B
SA-GFP
250
200
150
100
50
0
***
***
*
**
***
I-SceI: EV
Ctrl:
CTIP:
RIF1:
SHLD2: #1 #2
siRNA condition
C
siRNA condition
Ctrl
SHLD2
D
Ctrl
+ NCS (1hr)
SHLD2:
CtIP:
Ctrl:
NCS:
WB
46
32
58
46
p-RPA2 S4/S8
Tubulin
E
DR-GFP
125
100
75
50
25
0
**
*
I-SceI: EV
Ctrl:
BRCA1:
FAM35A: #1 #2
siRNA condition

Figure EV5.

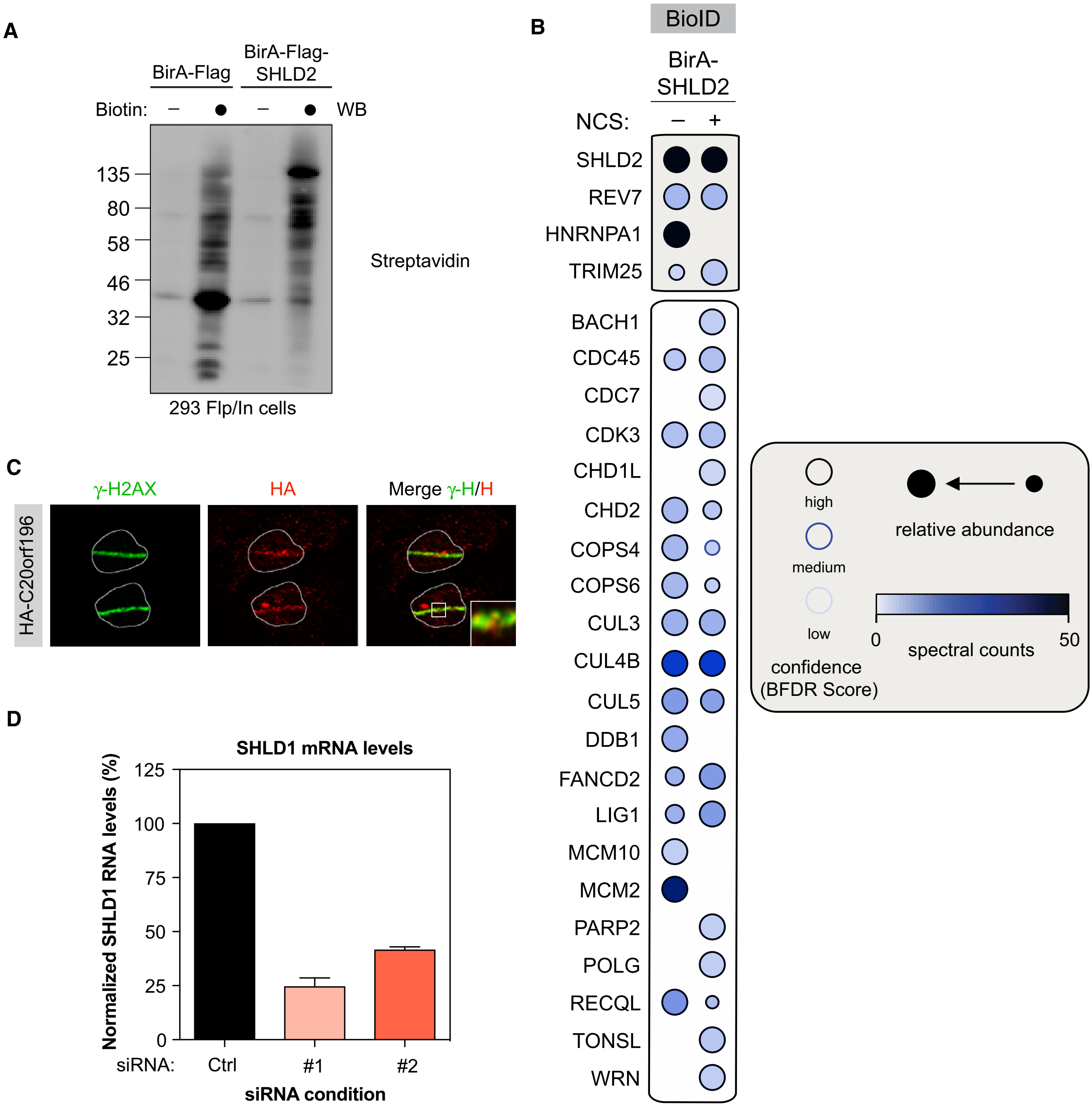

Figure EV6. Identification of SHLD2 interactome.

A HEK293-TREx cells transfected with a BirA-Flag or BirA-Flag-SHLD2 construct were tested for biotinylation following incubation with biotin as indicated. After lysis, samples were immunoblotted for streptavidin.

B Selected BioID SHLD2 results, shown as dot plots. The spectral counts for each indicated prey protein are shown as AvgSpec. Proteins were selected based on and iProphet probability of > 0.95, BFDR of < 0.05 and ≥ 10 peptide count. The circle size represents the relative abundance of preys over baits.

C U2OS cells stably expressing HA-SHLD1 were processed as previously described. Immunofluorescence against endogenous HA and γ-H2AX epitope was subsequently performed to monitor their accumulation at sites of damage. Shown are representative micrographs.

D EJ5-2OS cells were transfected with small interfering RNA against SHLD1 for 48 h, total RNA was isolated, cDNA was generated, and levels of SHLD1 were identified by qPCR. mRNA levels were normalized to mRNA levels of GAPDH.

 © 2018 The Authors
